# Impact of Temperature, Ethanol and Cell Wall Material Composition on Cell Wall-Anthocyanin Interactions

**DOI:** 10.3390/molecules24183350

**Published:** 2019-09-14

**Authors:** Cristina Medina-Plaza, Jordan W. Beaver, Larry Lerno, Nick Dokoozlian, Ravi Ponangi, Tom Blair, David E. Block, Anita Oberholster

**Affiliations:** 1 Department of Viticulture and Enology, University of California-Davis, 595 Hilgard Ln, Davis, CA 95616, USA; cmedinaplaza@ucdavis.edu (C.M.-P.); jwbeaver@ucdavis.edu (J.W.B.); lalerno@ucdavis.edu (L.L.); deblock@ucdavis.edu (D.E.B.); 2 E&J Gallo Winery, 600 Yosemite Blvd, Modesto, CA 95354, USA; nick.dokoozlian@ejgallo.com (N.D.); ravi.ponangi@ejgallo.com (R.P.); tom.blair@ejgallo.com (T.B.); 3 Department of Chemical Engineering, University of California-Davis, One Shields Avenue, Davis, CA 95616, USA

**Keywords:** anthocyanin, cell wall material, adsorption, desorption, phenolics, extractability, grape, red wine

## Abstract

The effects of temperature and ethanol concentration on the kinetics of anthocyanin adsorption and desorption interactions with five cell wall materials (CWM) of different composition were investigated. Using temperatures of 15 °C and 30 °C and model wine with ethanol concentrations of 0% and 15% (*v*/*v*) over 120 min, the adsorption and desorption rates of five anthocyanin-glucosides were recorded in triplicate. Small-scale experiments were conducted using a benchtop incubator to mimic a single berry fermentation. Results indicate that more than 90% of the adsorption occurs within the first 60 min of the addition of anthocyanins to CWM. However, desorption appears to occur much faster, with maximum desorption being reached after 30 min. The extent of both adsorption and desorption was clearly dependent not only on temperature and ethanol concentration but also on the CWM composition.

## 1. Introduction

Anthocyanins constitute a large family of plant polyphenols and are responsible for many of the fruit and floral colors observed in nature [1]. Anthocyanins are water-soluble pigments located in the grape skin vacuoles that, during the fermentation process, are released into the wine. It has been demonstrated that determining the amount of pigments present in the berries is not enough to estimate the concentration of anthocyanin in the final product [2]. This lack of correlation is mainly attributed to the interaction between the pigments and the skin cell walls during the extraction process [3,4]. Additionally, the adsorption of phenolics to solids in the fermentor after being released, such as grape skins and yeast hulls, has previously been demonstrated [5,6,7].

Previous studies have shown that the interaction between polyphenols and skin cell walls is dependent on the composition of the latter suggesting that specific cell wall constituents show different adsorption capacities for polyphenols [3,8,9,10,11]. In the case of anthocyanins, the cellulose content and the degree of methylation of the pectin have shown positive correlations with the adsorption capacity [4,12]. Moreover, some studies suggest that other components of the cell walls, such as proteins, can occupy binding sites resulting in overall lower anthocyanin adsorption [12].

Another factor that greatly influences the extraction of phenolics during wine fermentation has been shown to be the temperature at which the fermentation is performed. Previous research has shown that elevated fermentation temperatures (approaching 30 °C) produce finished wines that are more highly colored and have greater concentrations of pigmented polymers [13]. The increase in extracted phenolics at elevated temperatures has been accredited to two temperature related effects: an increased permeability of the hypodermal cells of the grape skins and an increase in the solubility of phenolics at higher temperature. It has also been shown that changes on the temperature can impact the physical structure of the cell wall material (CWM). It has been postulated that, at high temperatures, the cellulose structure opens up, potentially creating new sites and a faster exchange between the molecules [14]. Additionally, an increase in temperature can disrupt hydrogen bonds between the cell wall and the phenolics increasing its concentration in solution [15]. A second fermentation factor that is also likely to have a significant effect is the production of ethanol (EtOH) during fermentation. As the EtOH concentration increases during fermentation, the solubility of polyphenols, including the larger and more hydrophobic phenolics, will increase [16]. Moreover, a decrease in the polarity of the solution by the presence of ethanol can disrupt hydrophobic interaction, increasing the molecules in solution [15].

To the best of our knowledge, there is only one other study that has investigated the decrease in anthocyanin concentration over time when in contact with skin cell wall analogues [15]. No known study has analyzed the synergistic effect of EtOH and temperature on the adsorption of anthocyanins overtime as well as their influence on adsorption to different cell wall material components.

In this work, the effects of temperature and EtOH concentration on the kinetics of anthocyanin adsorption and desorption interactions were investigated with five different CWM compositions. Using temperatures of 15 °C and 30 °C and model wine with EtOH content of 0% and 15% over a time period of 120 min, the adsorption and desorption interactions of five anthocyanin-glucosides with CWM were analyzed.

## 2. Results and Discussion

### 2.1. Characterization of Cell Wall Material

Ash, lipid content, proteins, uronic acid, soluble polysaccharides, cellulose, Klason lignin, and non-cellulosic glucose of all the different CWMs isolated were determined (Table 1).

In all cases, the ash content was lower than 5%. This agrees with previous characterization of CWM from different cultivars [17] as the main components of CWM are cellulose, pectin, hemicellulose and lignin. CWM4 and CWM5 had slightly higher protein content due to the absence of the phenol wash during the isolation process. CWM2 exhibited a higher protein content than CWM1 and CWM3 potentially due to the linking of oligosaccharides to proteins [18]. CWM1 and CWM3, although lower than the other CWM preparations, still contained a significant amount of protein due the fact that the phenol buffer treatment only removes cytoplasmic proteins. Other types of proteins may exist within the structure of the CWM matrix such as glycoproteins and wall proteins [19]. Regarding lipid concentration, CWM3 and CWM5 presented the highest values due to the absence of the MeOH/chloroform extraction during the isolation process. As for soluble polysaccharide content, the CWM that were extracted with HEPES buffer (CWM1, CWM3, and CWM4) presented very low amounts. The small amounts found could be explained by the existence of glycolipids and glycoproteins on the CWM that were not removed during the different washes [20]. The difference in soluble polysaccharides between CWM2 and CWM5 may be the result of consecutive washings of CWM2 to remove other CWM components resulting in inadvertent removal of polysaccharides. The amounts of Klason lignin, cellulosic glucose and non-cellulosic glucose as well as uronic acids are comparable for all types of CWM analyzed, as they are not influenced by any of the extraction solvents used during the different isolation steps. The total polyphenolic content was less than 5% in all cases.

Our findings are in agreement with previous studies that characterized grape CWM [4,21] although this study is the only known investigation of the compositional impact of each CWM cleaning step.

### 2.2. Adsorption Kinetics

**Figure 1:**
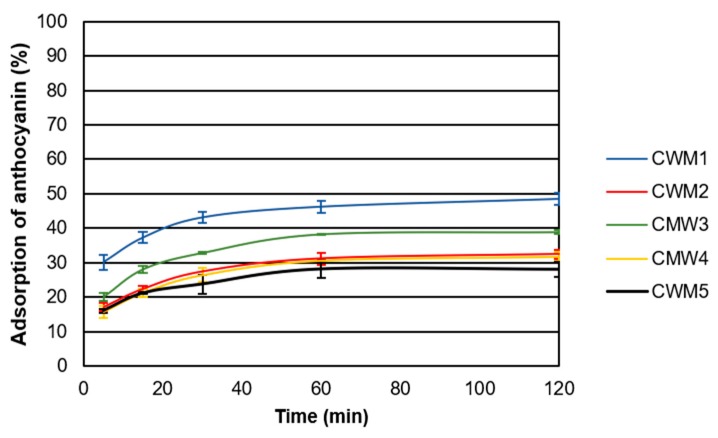
Adsorption kinetics for different types of CWM obtained at 15 °C in the presence of alcohol (15% EtOH) (*n* = 3). Error bars represent the standard deviation of replicate measurements (*n* = 3).

Figure 1 shows the adsorption kinetics of anthocyanins during experiments performed with different types of CWM at low temperature (15 °C) in the presence of alcohol (15% EtOH). Under these conditions, the percentage of anthocyanin molecules adsorbed onto CWM varied from 28% ± 2% to 48% ± 3%. Even though each type of CWM reached a different maximum adsorption percentage, the time to this maximum adsorption was comparable for all, reaching a plateau after 60 min. Small adsorption changes between 60 and 120 min were found to be significant for all treatments. Preliminary experiments were carried out over 420 min but no significant changes were observed after 120 min (data not shown). Previous studies investigating the binding of polyphenols to different cell wall components found similar trends with the most binding occurring in the first 30 min to 1 h of contact [12,15,21,22].

The adsorption capacity of CWM1 was larger than all others potentially due to the presence of more binding sites available for the anthocyanin molecules (Figure 1). Conversely, CWM5 showed the lowest percentage of adsorption of anthocyanins possibly attributable to the blocking of the binding sites by the different macromolecules that would have been removed with the additional cleaning steps present in other types of CWM. The composition of the different types of CWM suggests that proteins and polysaccharides have a larger impact on the adsorption process, potentially due to their larger concentration compared to that of lipids. In addition, polysaccharides and proteins are generally larger molecules than lipids. This may explain the adsorption differences found among CWM2, CWM3 and CWM4, with CWM3 exhibiting a larger anthocyanin adsorption ratio than CWM2 and CWM4. Larger molecules would likely occupy more space within the CWM matrix effectively decreasing the accessibility to the binding sites for anthocyanin molecules resulting in lower maximum adsorption percentages. This trend was consistently found for all the experiments performed. These results are in agreement with previous findings where polyphenols were found to be bound less to CWM in the presence of proteins due to potential blocking of binding sites [12].

The results showed that both temperature and EtOH concentration impacted anthocyanin adsorption. For each type of CWM, an increase in EtOH concentration and/or temperature increased the association of anthocyanin with molecules in solution rather than binding sites on the CWM. The large difference found between CWM1 and CWM 2–5 in the presence of alcohol and higher temperature (15% EtOH, 30 °C) could be attributed to expansion of the CWM matrix caused by the temperature and EtOH presence [23]. It has also been probed that changes on temperature and ethanol can modify the interactions occurring between CWM and anthocyanins such as hydrogen bonds and coulombic interactions [10,24]. The absence of the macromolecules interwoven with the CWM network (CWM1) makes this CWM more sensitive to EtOH and temperature changes leading to an opening up of the structure making more binding sites available.

Regarding the type of anthocyanin, no differences in binding were found between non-acetylated and acetylated anthocyanin. Nevertheless, the detailed anthocyanin profile showed that delphinidin-3-glucoside and petunidin-3-glucoside had a larger percentage adsorbed compared to the rest of the molecules analyzed at all the conditions analyzed. The adsorption percentage of delphinidin 3-glucoside ranged from 20% to 85% depending on the experimental conditions, and malvidin 3-glucoside adsorption percentage ranged from 10% to 70%. This trend was found for all the types of CWM suggesting that the presence of hydroxyl groups on the anthocyanin contribute to the potential hydrogen bonding between the anthocyanin molecules and the CWM polysaccharides that influence adsorption kinetics. Similar to these findings, previous studies found that non-acetylated and acetylated anthocyanin showed similar behavior in the presence of skin CWM [12,15,24]. Additionally, Vasserot et al. obtained similar results regarding polarity (hydroxylation on the B ring) on the study of adsorption of five monoglycoside anthocyanins onto yeast CWM in the presence of alcohol [6]. Table 2 shows the percentage of adsorption of individual anthocyanin species onto CWM1 under all the conditions analyzed. In the absence of EtOH the order of anthocyanins was: delphinidin-3-glucoside, petunidin-3-glucoside, malvidin-3-glucoside, malvidin-3-acetyl-glucoside and peonidin-3-glucoside. However, when EtOH concentration increases to 15%, the order of anthocyanins changes to delphinidin-3-glucoside, petunidin-3-glucoside, peonidin-3-glucoside, malvidin-3-glucoside and malvidin-3-acetyl-glucoside. Adsorption fluctuations could be due to the disruption of hydrogen bonds by EtOH [15]. Additionally, the decrease in polarity of the solution in the presence of EtOH increased the concentration of the less polar molecules in solution. The presence of EtOH did not have a large impact on the adsorption process, this could be potentially due to the fact that the maximum concentration tested was 15%. Furthermore, the order of anthocyanin adsorption was not impacted by temperature changes between 15 and 30 ˚C. This trend was observed for all the different CWM matrixes tested.

**Figure 2:**
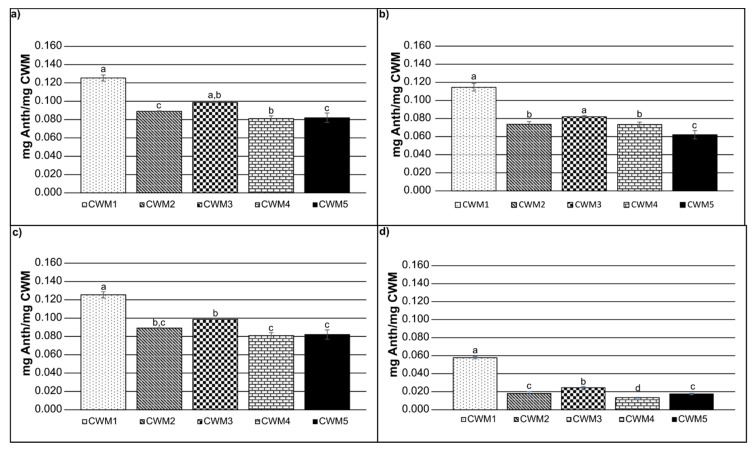
Anthocyanin adsorption values reached after 120 min for: (**a**) 0% EtOH and 15 °C, (**b**) 15% EtOH and 15 °C, (**c**) 0% EtOH and 30 °C, (**d**) 15% EtOH and 30 °C; Error bars represent the standard deviation of replicate measurements (*n* = 3). Significance was determined using analysis of variance (ANOVA).

In order to consider the ratio of the CWM to anthocyanin molecules in solution, weight per weight calculations were performed. Figure 2 shows the amount of anthocyanins (mg) adsorbed per mg of CWM after 120 min. The results indicate that for all the conditions CWM1 presented the highest anthocyanin adsorption value, while CWM5 showed the lowest. As stated before, CWM3 tended to reach a larger adsorption ratio than CWM2 and CWM4 likely due to the smaller size of the lipids and the absence of larger molecules blocking the binding sites, although it was not significant in all cases. Moreover, lipids are more significantly influenced by temperature and EtOH concentrations, modifying their fluidity and likely making binding sites more available when the EtOH content or the temperature increases compared to other macromolecules [25].

Significant differences in the binding response between anthocyanin and the CWM at different conditions were determined from triplicate experiments using a multi-way analysis of variance (MANOVA). The results indicated that all the variables (temperature, cell wall composition and EtOH concentration) have a significant impact on the adsorption process (*p* < 0.001).

It has been observed that anthocyanin molecules can undergo thermal degradation by breaking the *O*-glycosidic bond [26,27,28]. In this study, the potential presence of break-down products produced by the degradation of anthocyanins was investigated by means of LC-DAD-MS/MS. In all samples, all screened break-down compounds fell below the LOD indicating changes in anthocyanin concentration were due to adsorption. This could be due to the fact that 30 °C is a low temperature to breakdown the short time period of the experiment (2 h).

### 2.3. Desorption Kinetics

**Figure 3:**
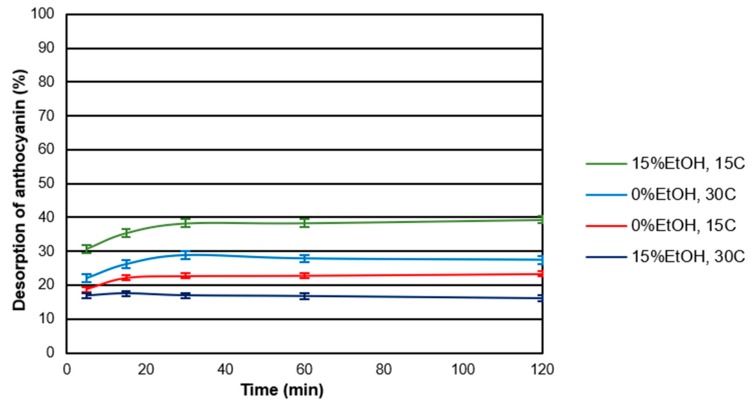
Desorption kinetics for CWM2 at all the experimental conditions (*n* = 3). Error bars included.

Desorption assays were performed under the same sets of temperature and EtOH as those for the adsorption experiments. The rates of the desorption process were faster than adsorption reaching a plateau within the first 30 min. Figure 3 shows the kinetics of desorption for CWM2 at all the conditions tested. As can be observed, the desorption kinetics depended not only on the conditions of the experiment but also on the amount of anthocyanin initially adsorbed onto the CWM. Concerning the type of anthocyanin, delphinidin-3-glucoside and petunidin-3-glucoside showed the lowest percentage of desorption suggesting the breakdown of hydrophobic interactions by the solvent prior to hydrogen bonds. Similar trends were found for the other CWMs studied.

Table 3 shows the amount of anthocyanin molecules adsorbed at the beginning of the desorption experiment, the amount released after 120 min and the percentage desorbed after 120 min for each of the experiments performed. At low temperature, the presence of alcohol resulted in an increase in the desorption percentage likely due to the disruption of the hydrophobic interactions [29] or an increase in the solubility of anthocyanins in solution. A similar trend was observed when the temperature was increased in the absence of alcohol. However, at a higher temperature in the presence of EtOH (15% *v*/*v*) this trend was not noted (increase on the desorption), potentially due to the expansion of the CWM [23] and the low amount adsorbed of anthocyanin adsorbed under these conditions.

Follow up experiments performed with CWM with the same amount of anthocyanin adsorbed indicated that both, temperature and EtOH concentration increase the desorption rate with temperature having a larger impact (data not shown).

To better understand the types of interactions taking place during adsorption it is important to point out that at the working pH, approximately 18% of the anthocyanin molecules were positively charged [30], while the CWM fibers (mainly cellulose, hemicellulose and pectin derivates) have been shown to have a negative surface charge. The results suggest the presence of different types of interactions between the CWM and the anthocyanin molecules. The existence of a base layer with the strongest interactions (coulombic interactions) between the anthocyanin and the CWM cellulose/pectin network, that will increase at higher temperatures due to the expansion of the CWM fibers, has been previously reported [10]. Additionally, hydrogen bonding between the hydroxyl groups of anthocyanins and the oxygen atoms of the cross-linked ether bonds of sugars present in the CW polysaccharides [24] as well as hydrophobic interactions take place. Moreover, π-π interactions between anthocyanin molecules can form anthocyanin self-association complexes, which can potentially stack on to the CWM. CWM is also a complex porous structure, which may trap molecules in solution.

The desorption results suggest that the amount of anthocyanin molecules released from the CWM depends on the strength of their interactions. Unlike the adsorption experiments, no clear trends on the desorption process were found depending on the CWM composition, although the quantity of molecules adsorbed was found to be dependent on CWM composition. The presence of the different macromolecules on the CWM modify the availability of the binding sites thus controlling the amount of anthocyanin adsorbed. Adsorption results suggested that anthocyanins do not interact with the macromolecules (soluble polysaccharides, cytoplasmic proteins and lipids) within the CW network and only with the polysaccharide network (primarily cellulose, pectin and hemicellulose) itself. Thus, the macromolecule composition of the CWM does not have a direct impact on the desorption process, explaining similar desorption from the different types of CWM.

## 3. Materials and Methods

### 3.1. Reagents

Acetone (reagent grade), acetonitrile (HPLC grade), methanol (MeOH) (reagent grade), hydrochloric acid (HCl) (37%, reagent grade), trifluoroacetic acid (TFA) (HPLC grade), formic acid (HPLC grade), sulfuric acid (96% reagent grade), diethyl ether (ACS reagent, 99%), phenol (reagent grade), L-ascorbic acid (molecular biology grade), bovine serum albumin (BSA), HEPES buffer, phenol solution equilibrated with 10 mM Tris HCl, potassium bitartrate (99%), (+)-catechin hydrate (98%), (−)-epicatechin (90%), p-coumaric acid (98%), ferulic acid (99%), caffeic acid (98%), quercetin (95%), gallic acid monohydrate (99%), syringic acid (98%), vanillic acid (97%), 4-hydroxybenzoic acid (99%), 3,4-dihydroxybenzoic acid (99%), sulfamic acid (99.9%), 3-phenylphenol (85%), sodium tetraborate (99%), d-galacturonic acid (97%), quercetin-3-glucoside (98.5%), and Toyopearl HW-50F size exclusion media were purchased from Sigma Aldrich (St. Louis, MO, USA). Bovine albumin standard solution (2.0 mg/mL) and Coomassie protein assay reagent were purchased from Thermo scientific (Waltham, MA, USA). Orthophosphoric acid (88%) (HPLC grade) and sodium hydroxide (ACS grade) were purchased from Fisher Scientific (Pittsburgh, PA, USA). Malvidin-3-*O*-glucoside (95%) was purchased from Extrasynthese (Genay, France). Koptec brand ethanol (95%) was purchased from Decon Laboratories, Inc. (King of Prussia, PA, USA). Deionized water was prepared in-house to a final purity of 18.2 MΩ.

### 3.2. Instrumentation

An Agilent 1260 Infinity HPLC (Agilent Technologies, Santa Clara, CA, USA) equipped with a diode array detector and an Agilent 6430 triple quadrupole mass spectrometer was used to analyze the adsorption and desorption samples as well as the purity of the anthocyanin extract. Instrument control and data analysis were performed using MassHunter (B.08.00) and Agilent CDS ChemStation (Rev. B.04.03) software, respectively.

### 3.3. Isolation of Cell Wall Material

CWM was isolated from Thompson Seedless grape skins due to its lower phenolic content as well as availability year-round. CWM was prepared using a modified method from Vidal et al. [16]. Skins were ground under liquid nitrogen using an analytical mill (A11 Basic, IKA^®^ Works, Inc., Wilmington, NC, USA) and incubated overnight in 70% *v/v* acetone at 4 °C to remove polyphenolic content. The solids were vacuum filtered (Whatman™ 1001–125 Grade 1 Qualitative Filter Paper, Diameter: 12.5 cm, Pore Size: 11 µm) and washed with 70% acetone until clear, followed by Milli-Q water (resistivity of 18.2 MΩ·cm; Millipore Corporation, Billerica, MA, USA). Subsequent residues were extracted with 40 mM HEPES buffer (pH 7) for one hour at room temperature (RT) to eliminate soluble polysaccharides. The HEPES-insoluble skin material was washed with Milli-Q water followed by 100% acetone and then treated for 30 min with phenol buffer (5 M in Tris-HCl, pH 7.5) at RT to remove cytoplasmic proteins [31]. The remaining CWM was vacuum filtered, washed with 80% *v/v* EtOH followed by acetone and then incubated for 30 min in MeOH/chloroform (1:1 *v*/*v*) to remove residual lipids. CWMs of different compositions were obtained by eliminating one of the washes from the process as defined in Table 4. CWM1 underwent all the washes, CWM2 had soluble polysaccharides left as it was not washed with the HEPES buffer, CWM3 had lipids left as it was not washed with MeOH/chloroform, while CWM4 had proteins left due to no phenol wash and finally CWM5 had only polyphenolics removed as it only went through the acetone wash. Finally, the CWM was dried under vacuum, passed through a 500 μm mesh and stored at –20 °C until needed.

### 3.4. Isolation of Anthocyanins

Anthocyanins were obtained from Cabernet Sauvignon grape skins. Cabernet Sauvignon was chosen as it has a large concentration of anthocyanin and is an important variety in CA. Eighty grape skins (8 g approximately) were ground (T18 digital ULTRA-TURRAX^®^, IKA^®^ Works, Inc., Wilmington, NC, USA) and incubated overnight at 4 °C with 50% acidified MeOH (0.1% *v/v* TFA) in a 1:10 *w/v* ratio. The methanolic extractions were purified by means of low-pressure chromatography using Toyopearl HW-50F as the stationary phase with a bed volume of 500 mL. The loaded sample was washed with two bed volumes of aqueous 0.1% TFA solution to eliminate sugars and salts. Anthocyanins were eluted using 30% acidified MeOH (0.1% *v/v* TFA). The eluate was concentrated under reduced pressure and the purity was determined using a RP-HPLC method previously published [32]. The purity of the anthocyanin material was always over 99%. The extract was lyophilized and stored at −80 °C. The process was repeated until enough anthocyanin material was isolated. The composition of the final anthocyanin extract was: 4.7% delphinidin-3-glucoside, 4.7% petunidin-3-glucoside, 4.6% peonidin-3-glucoside, 68.3% malvidin-3-glucoside, 17.70% malvidin-3-acetylglucoside.

### 3.5. Cell Wall Material Characterization

#### 3.5.1. Carbohydrate Composition and Ash Content

Non-cellulosic content was determined colorimetrically by means of the phenol-sulfuric method [19] after digestion of one gram of CWM with 1M sulfuric acid for 2.5 h at 100 °C. Soluble sugars were determined spectrophotometrically using the phenol-sulfuric assay [33] after the extraction of one gram of CWM with 40 mM HEPES buffer for one hour at RT [17]. Cellulose was determined as glucose concentration according to Lurie et al. [34] and using the phenol-sulfuric method for its spectrometric determination. Klason lignin was analyzed gravimetrically as acid-insoluble residue [17]. Ash content of CWM was gravimetrically determined by overnight heating at 550 °C [35].

#### 3.5.2. Lipid Analysis

CWM lipids were extracted overnight using a Soxhlet apparatus with diethyl ether (45 °C). Extracts were dried, and lipid content was determined gravimetrically.

#### 3.5.3. Protein Analysis

The protein content of CWM was determined using the Bradford assay [36] after digestion of the sample with 1M NaOH (10 min, 100 °C). BSA solution was used as standard to calibrate the analysis with standards ranging from 0 to 2000 μg/mL in concentration. Protein content was expressed as mg BSA/g CWM.

#### 3.5.4. Uronic Acid Analysis

Uronic acids were determined in the sulfuric acid hydrolysate by the colorimetric 3,5-dimethylphenol assay. [37] Pure galacturonic acid was used as standard. Uronic acid content was expressed as mg anhydrous galacturonic acid/g CWM.

#### 3.5.5. Phenolic Content

Phenolic content of CWM was determined colorimetrically [38] after extraction with 1M NaOH (100 °C, 10 min) using pure gallic acid as standard. Phenolic content was expressed as mg gallic acid/g CWM.

### 3.6. Adsorption Kinetics of Isolated Anthocyanin onto Cell Wall Material

The effects of EtOH concentration, temperature and the composition of the CWM on the kinetics of anthocyanin adsorption onto CWM were investigated using a full factorial design with temperatures of 15 °C and 30 °C and EtOH content of 0% and 15% (*v*/*v*) for all the different CWMs isolated. The experiments simulated a single berry fermentation environment [39]. Ten milligrams of isolated CWM (CWM1-CWM5) equilibrated in 0.3 mL of model wine (5 g/L potassium bitartrate, 0.1% *w*/*w* ascorbic acid, pH 3.5), with or without alcohol, were put in contact with 1 mL of anthocyanin solution reaching a final concentration of 2 mg/mL. Contact between the CWM particles and the anthocyanin molecules was maximized by using a rotator (H5600 Revolver™ Adjustable Lab Rotator, Labnet International, Inc., Edison, NJ, USA). The supernatant was sampled (10 µL aliquots) after 5, 15, 30, 60, and 120 min. Samples were diluted ten times in 0.1% aqueous HCl solution and stored at −80 °C until analysis. All the experiments were carried out in triplicate. A control of the anthocyanin solution without CWM was used to monitor potential side reactions and to calculate adsorption rate by comparison between the blanks and the trials.

### 3.7. Desorption Kinetics Experiments

Desorption experiments were carried out after the completion of the adsorption study. Samples were centrifuged at 15,000 rpm for 5 min and the supernatant was removed. One ml of model wine (5 g/L potassium bitartrate, 0.1% *w*/*w* ascorbic acid, pH 3.5) containing either 0% or 15% EtOH, was added to the CWM with the anthocyanin adsorbed. The test tubes were incubated under the same conditions as used in the adsorption experiments. The analysis was performed in triplicate and 10 μL aliquots were sampled after 5, 15, 30, 60, and 120 min. Samples were diluted ten times in 0.1% HCl *v/v* solution to quench any reaction and stored at −80 °C until analyzed.

### 3.8. Determination of Anthocyanin and Break Down Products by LC-DAD-MS

Individual anthocyanins and their break down products were quantified by LC-DAD-MS/MS using a Poroshell-120 C18 column (Agilent technologies, 2.7 μm, 120 Ǻ, 2.1 × 50 mm) protected with a guard cartridge (SecurityGuard™ ULTRA cartridges UHPLC, 2 μm, Phenomenex, Torrance, CA, USA). The mobile phases were solvent A, 7.5% (*v*/*v*) formic acid in water and solvent B, 0.1% (*v*/*v*) formic acid in acetonitrile. The following linear gradient was used for solvent A (with solvent B making up the remainder): 97% at 0 min; 77% at 4 min; 20% at 4.5 min; then returning to the starting conditions at 7−12 min, 97%. A flow rate of 0.55 mL/min was used with a column temperature of 40 °C. Anthocyanin concentrations were determined using an external calibration curve of malvidin-3-glucoside and were calculated as the sum of all the anthocyanins detected expressed as malvidin-3-glucoside. Anthocyanin molecules were analyzed using DAD signal whereas break-down products were determined by means of mass spectrometry MS analysis was performed in positive ionization mode using an ESI source. N_2_ was used as the nebulizer and drying gas at flows of 35 psi and 12 l/min respectively. Parameters were set to: capillary voltage of 3.5 kV and fragmentor voltage of 90 V; drying gas temperature 350 °C, respectively. Detection was in multiple reaction monitoring (MRM) mode with the following transitions for each break down product: syringic acid (*m*/*z* 125 > 155, 2.050 min), methyl gallic acid (*m*/*z* 139 > 167, 0.780 min), gallic acid (*m*/*z* 81 > 127, 0.413 min), vanillic acid (*m*/*z* 65 > 125, 1.380 min) and protocatechuic acid (*m*/*z* 111 > 153, 0.595 min). External calibration curves based on the corresponding standard were used to determine the concentration of individual phenolics.

### 3.9. Statistical Analysis

Significant differences in the processes and conditions were determined from triplicate experiments by means of multi-way analysis of variance (MANOVA) (*p* < 0.001) using XLStat (Microsoft Office, version 2018.5.53172).

## 4. Conclusions

Interactions between anthocyanin molecules and skin CWM can occur spontaneously and rapidly. For all the experiments performed the maximum adsorption was reached after 60 min, suggesting no influence of the composition of the CWM on the rate of adsorption. However, the presence of different macromolecules (polysaccharides, proteins or lipids) on the CWM does result in the modification of the binding capacity of the CWM. The amount of adsorbed molecules was found to be positively influenced by the absence of large macromolecules blocking the binding sites, attaining the maximum percentage of adsorption with the cleanest CWM (CWM1). Both temperature and alcohol percentage had a significant impact on adsorption. All the experiments showed that an increase in temperature and ethanol produces a decrease in the adsorption percentage potentially due to the increase of the solubility of the pigments in the model wine. Anthocyanin polarity appeared to be important as the more polar molecules showed a higher percentage of adsorption. Desorption was mostly influenced by temperature and EtOH increasing the desorption rate—no trends were found regarding CWM composition.

The results suggest the presence of different types of interactions between the CWM and the anthocyanin molecules. The existence of a base layer with the strongest interactions (coulombic interactions) between the anthocyanin and the CWM cellulose/pectin network has been previously reported [10]. Additionally, hydrogen bonding between the hydroxyl groups of pigments and the oxygen atoms of the cross-linked ether bonds of sugars present in the CW polysaccharides [24] as well as hydrophobic interactions take place. Additionally, concentration related π-π interactions between anthocyanin molecules can potentially form anthocyanin complexes, which can potentially stack on to the CWM. CWM is also a complex porous structure, which may trap molecules in solution.

Our findings are in good agreement with the phenomena routinely observed in wineries where wines with higher alcohol content or fermented at higher temperatures extract more phenolics and color [26,40,41]. This work shows that a high concentration of phenolics in wine depends not only on grape phenolic composition but also on temperature and EtOH conditions during fermentation as well as adsorption/desorption interactions of the phenolics with solids in the fermentor. Additionally, differences in the composition of grape CWM could directly affect both the release and adsorption processes, leading to a different final wine phenolic profile.

## Figures and Tables

**Table 1:** CWM composition. CWM 1–5 as defined in materials and methods. Different letters indicate significantly differences within a column using Fisher test *p* < 0.05 (*n* = 3).

CWM	Protein (mg BSA/g CWM)	Lipids (mg/g CWM)	Soluble Polysaccharides (mg/g CWM)	Non-Cellulosic Glucose (mg/g CWM)	Cellulosic Glucose (mg/g CWM)	Lignin (mg/g CWM)	Uronic Acid (mg/g CWM)	Total Polyphenolic Content (mg Gallic Acid/g CWM)	Ash (mg/g CWM)
**CWM1**	51.29 ± 1.14 ^c^	18.51 ± 2.38 ^bc^	3.00 ± 0.04 ^c^	84.17 ± 0.59 ^b^	51.23 ± 1.82 ^c^	407.23 ± 10.51 ^ab^	0.22 ± 0.00 ^a^	0.035 ± 0.000 ^c^	15.43 ± 2.08 ^e^
**CWM2**	54.91 ± 1.74 ^b^	13.62 ± 3.46 ^c^	5.49 ± 0.35 ^b^	94.23 ± 5.42 ^b^	55.06 ± 0.80 ^b^	418.42 ± 19.70 ^ab^	0.21 ± 0.01 ^a^	0.043 ± 0.003 ^b^	19.34 ± 3.27 ^c^
**CWM3**	48.86 ± 0.87 ^c^	33.61 ± 5.04 ^a^	3.04 ± 0.14 ^b^	87.44 ± 2.30 ^b^	54.10 ± 2.03 ^d^	408.52 ± 40.68 ^ab^	0.22 ± 0.01 ^a^	0.049 ± 0.000 ^a^	34.24 ± 5.01 ^b^
**CWM4**	57.96 ± 2.36 ^a^	15.08 ± 2.34 ^bc^	2.33 ± 0.20 ^bc^	111.29 ± 2.86 ^a^	57.04 ± 1.12 ^a^	371.96 ± 43.47 ^b^	0.20 ± 0.01 ^a^	0.043 ± 0.002 ^b^	15.96 ± 2.33 ^d^
**CWM5**	56.46 ± 1.73 ^ab^	24.23 ± 5.18 ^ab^	10.17 ± 0.88 ^a^	111.13 ± 7.06 ^a^	55.90 ± 0.29 ^ab^	475.54 ± 27.83 ^a^	0.22 ± 0.01 ^a^	0.021 ± 0.002 ^d^	49.41 ± 6.36 ^a^

**Table 2:** Percentage of adsorption of individual anthocyanins at 120 min for CWM1. Delph-delphinidin-3-glucoside; Pet-petunidin-3-glucoside; Peo-peonidin-3-glucoside; Malv-malvidin-3-glucoside; Malv-acet-malvidin-3-acetyl-glucoside.

Condition	Delph (%)	Pet (%)	Peo (%)	Malv (%)	Malv-Acet (%)
0%EtOH; 15 °C	78.57	75.94	32.60	60.55	54.79
15%EtOH; 15 °C	68.89	65.10	42.04	38.34	29.08
0%EtOH; 30 °C	69.27	63.28	47.55	29.68	47.43
15%EtOH; 30 °C	23.58	20.73	16.81	15.06	16.16

**Table 3:** Values of the mass adsorbed and desorbed for each of the conditions analyzed after 120 min (*n* = 3). Percentage of desorption reached for each condition at 120 min. RSD < 5% for all the measurements performed.

EtOH (%)	T (°C)	CWM	Adsorbed (mg Anth/mg CWM)	Desorbed (mg Anth/mg CWM)	Percentage Desorbed (%)
0	15	CWM1	0.160	0.029	18.12
0	15	CWM2	0.155	0.028	18.06
0	15	CWM3	0.172	0.025	14.53
0	15	CWM4	0.159	0.025	15.72
0	15	CWM5	0.148	0.025	16.89
15	15	CWM1	0.128	0.037	28.90
15	15	CWM2	0.108	0.043	39.81
15	15	CWM3	0.114	0.035	30.70
15	15	CWM4	0.103	0.035	33.98
15	15	CWM5	0.102	0.030	29.41
0	30	CWM1	0.137	0.024	17.51
0	30	CWM2	0.128	0.037	28.90
0	30	CWM3	0.132	0.025	18.94
0	30	CWM4	0.127	0.030	23.62
0	30	CWM5	0.125	0.029	23.20
15	30	CWM1	0.112	0.014	12.50
15	30	CWM2	0.100	0.013	13.00
15	30	CWM3	0.104	0.016	15.38
15	30	CWM4	0.093	0.013	13.97
15	30	CWM5	0.092	0.012	13.04

**Table 4:** types of CWM prepared and × indicate that the step was not performed.

Type of CWM	70% Acetone	Buffer HEPES	Phenol Solution	MeOH/Chloroform (1:1 *v*/*v*)	Composition
CWM1	√	√	√	√	Clean cell wall material
CWM2	√	×	√	√	CWM1 + Soluble Polysaccharides
CWM3	√	√	√	×	CWM1 + Lipids
CWM4	√	√	×	√	CWM1 + Cytoplasmic proteins
CWM5	√	×	×	×	“Crude” cell wall material
